# Lack of association between *PER3* variable number tandem repeat and circadian rhythm sleep–wake disorders

**DOI:** 10.1038/s41439-018-0017-7

**Published:** 2018-07-12

**Authors:** Akiko Hida, Shingo Kitamura, Hiroshi Kadotani, Makoto Uchiyama, Takashi Ebisawa, Yuichi Inoue, Yuichi Kamei, Kazuo Mishima

**Affiliations:** 1 Department of Sleep-Wake Disorders, National Institute of Mental Health, National Center of Neurology and Psychiatry, Tokyo, 187-8553 Japan; 2 Department of Sleep and Behavioral Sciences, Shiga University of Medical Science, Shiga, 520-2192 Japan; 3 Department of Psychiatry, Nihon University School of Medicine, Tokyo, 173-8610 Japan; 4 Yokohama Clinic for Psychosomatic Medicine and Psychiatry, Medical Corporation Warakukai, Kanagawa, 220-0004 Japan; 5 Department of Somnology, Tokyo Medical University, Tokyo, 160-8402 Japan; 6 Yoyogi Sleep Disorder Center, Tokyo, 151-0053 Japan; 7 Department of Laboratory Medicine, National Center Hospital, National Center of Neurology and Psychiatry, Tokyo, 187-8551 Japan

## Abstract

Circadian rhythm sleep–wake disorders (CRSWDs) are characterized by disturbed sleep–wake patterns. We genotyped a *PER3* variable number tandem repeat (VNTR) in 248 CRSWD individuals and 925 controls and found no significant association between the VNTR and CRSWDs or morningness–eveningness (diurnal) preferences in the Japanese population. Although the VNTR has been associated with circadian and sleep phenotypes in some other populations, the polymorphism may not be a universal genetic marker.

Circadian clocks regulate daily behavioral and physiological rhythms, such as sleep and wakefulness^1^. The molecular mechanism of the circadian clock system involves transcription–translation-negative feedback loops of multiple clock genes, including *ARNTL* (also known as *BMAL1*), *Casein Kinase I* (*CKI*), *CLOCK*, *CRY*, and *PER*. The BMAL1 and CLOCK proteins form heterodimers and activate transcription of *CRY* and *PER*. The CRY and PER proteins gradually accumulate in the cytoplasm, and CRY, PER, and CKI proteins form complexes that translocate to the nucleus. These protein complexes interact with BMAL1–CLOCK heterodimers, thereby inhibiting transcription of the *CRY* and *PER* genes^2^. Circadian rhythm sleep–wake disorders (CRSWDs) are defined by persistent or recurrent disturbed sleep–wake patterns and are thought to result from impairment of the circadian clock system^3^. CRSWDs consist of several subtypes, including delayed sleep–wake phase disorder (DSWPD) and non-24-hour sleep–wake rhythm disorder (N24SWD). DSWPD is characterized by significantly delayed sleep timing, while N24SWD is characterized by sleep timing that progressively delays each day in normal real-life settings. Additionally, circadian and sleep characteristics vary greatly among individuals^4^. A number of studies have shown that genetic factors significantly contribute to individual differences in daily activity/sleep time, known as morningness–eveningness preferences (or diurnal preferences), and sleep phenotypes^5^. The *PER3* gene has a variable number tandem repeat (VNTR) consisting of either 4 or 5 repeated 54-bp sequences encoding 18 amino acids^6^. The 4-repeat allele of the VNTR has been associated with extreme evening preference and DSWPD^6,7^, while the 5-repeat allele of the VNTR has been associated with extreme morning preference and greater sleep propensity^7,8^. However, the frequency of the VNTR polymorphism differs among various populations^9,10^ and has not been examined in a large Japanese cohort of CRSWD patients and controls. In this study, we genotyped the *PER3* VNTR polymorphism in 180 individuals with DSWPD, 68 individuals with N24SWD, and 925 controls and tested the VNTR polymorphism for associations with CRSWD phenotypes in the patient group and with diurnal preferences in the control group.

The study population consisted of 180 DSWPD individuals (109 men and 71 women; mean ± SD age: 26.71 ± 9.29 years), 68 N24SWD individuals (48 men and 20 women; mean ± SD age: 26.63 ± 9.74 years), and 925 controls (274 men and 651 women; mean ± SD age: 36.45 ± 12.10 years). Patient subjects and controls had been examined in an earlier study of ours^11^. All participants were unrelated, sighted Japanese men and women who were recruited at medical and research institutes in Japan. None of the controls had a history of sleep disorders or psychosis. The DSWPD and N24SWD patients were diagnosed by trained psychiatrists according to the International Classification of Sleep Disorders, Third Edition (ICSD-III, 2014)^12^. Diurnal preferences were assessed by the Japanese version of the Horne–Östberg Morningness-Eveningness Questionnaire (MEQ)^13^ in our previous study^11^. The 925 controls consisted of 245 morning types (79 men and 166 women; mean ± SD age: 36.23 ± 12.19 years), 594 intermediate types (163 men and 431 women; mean ± SD age: 36.86 ± 12.05 years), and 86 evening types (32 men and 54 women; mean ± SD age: 37.60 ± 10.69 years). The protocol was approved by the respective institutional ethical review boards. All subjects provided written informed consent. The present study was conducted according to the principles of the Declaration of Helsinki.

The 4- or 5-repeat allele of the *PER3* VNTR was assessed by polymerase chain reaction (PCR). The primers used to amplify the DNA fragments containing 4 or 5 repeated 54-bp sequences were as follows: 5′-CAAAATTTTATGACACTACCAGAATGGCTGAC-3′, 5′-AACCTTGTACTTCCACATCAGTGCCTGG-3′. The PCR reaction was carried out in a 10-µl mixture containing AmpliTaq Gold 360 Master Mix and 360 GC Enhancer (Thermo Fisher Scientific, CA, USA), 1 µM of each primer, and 10 ng of genomic DNA and run in the GeneAmp PCR System 9600 (Thermo Fisher Scientific, CA, USA). Amplification conditions were as follows: 1 cycle of 95 °C for 5 min, 35 cycles of 95 °C for 30 s, 60 °C for 30 s, 72 °C for 30 s, and 1 cycle of 72 °C for 7 min. The PCR fragments were subject to electrophoresis in a 3% agarose gel.

Table 1 shows the genotypic and allelic distribution of the *PER3* VNTR in controls, DSWPD individuals, and N24SWD individuals. Homozygosity for the 4-repeat allele *PER3*-(4/4) is the most common (63.89–70.59 %), and homozygosity for the 5-repeat allele *PER3*-(5/5) is the least common (0–5 %), with the 4-repeat allele having higher prevalence (79.44–85.29 %) than the 5-repeat allele (14.71–20.56 %) in the control, DSWPD, and N24SWD groups. The allele frequency of the *PER3* VNTR was compared among the three groups, and no significant difference was identified in the VNTR frequency (*χ*^2^ = 3.0608, *P* = 0.2165). The *PER3* VNTR was not associated with CRSWD phenotypes in our study samples. We then assessed the association of the *PER3* VNTR with diurnal preferences (Table 2). Similarly, *PER3*-(4/4) is the most common polymorphism (62.79–70.88 %), *PER3*-(5/5) is the least common polymorphism (1.63–5.81 %), and the 4-repeat allele is more prevalent (78.49–83.67 %) than the 5-repeat allele (16.33–21.51 %) in the morning-, intermediate-, and evening-type groups. The VNTR alleles did not differ among these three types (*χ*^2^ = 3.1694, *P* = 0.205). No significant association was identified between the *PER3* VNTR and diurnal preferences in our control samples. Age-adjusted MEQ scores were 52.93 ± 7.86 (mean ± SD) for *PER3*-(4/4), 53.56 ± 8.57 for *PER3*-(4/5), and 50.02 ± 8.06 for *PER3*-(5/5). There was no difference in age-adjusted MEQ scores among the three genotypes (*F*(2, 922) = 2.171; *P* = 0.115).

**Table 1:** Genotype and allele frequency of the *PER3* VNTR in the control, DSWPD and N24SWD groups

Group	*N*	Genotype [*N* (%)]			Allele [*N* (%)]		*χ* ^2^	*P*
		4/4	4/5	5/5	4	5		
Control	925	635 (68.65)	260 (28.11)	30 (3.24)	1530 (82.70)	320 (17.30)	3.0608	0.2165
DSWPD	180	115 (63.89)	56 (31.11)	9 (5.00)	286 (79.44)	74 (20.56)		
N24SWD	68	48 (70.59)	20 (29.41)	0 (0.00)	116 (85.29)	20 (14.71)		

*N* number, *4/4* homozygosity for the 4-repeat allele, *4/5* heterozygosity, *5/5* homozygosity for the 5-repeat allele, *P* *P* value for the difference in allele frequency among the control, DSWPD, and N24SWD groups

**Table 2:** Genotype and allele frequency of the *PER3* VNTR in the morning, intermediate, and evening types

Type	*N*	Genotype [*N* (%)]			Allele [*N* (%)]		*χ* ^2^	*P*
		4/4	4/5	5/5	4	5		
Morning	245	160 (65.31)	81 (33.06)	4 (1.63)	401 (81.84)	89 (18.16)	3.1694	0.205
Intermediate	594	421 (70.88)	152 (25.59)	21 (3.54)	994 (83.67)	194 (16.33)		
Evening	86	54 (62.79)	27 (31.40)	5 (5.81)	135 (78.49)	37 (21.51)		

*N* number, *4/4* homozygosity for the 4-repeat allele, *4/5* heterozygosity, *5/5* homozygosity for the 5-repeat allele, *P* *P* value for the difference in allele frequency among the morning, intermediate, and evening types

We found no significant association of the *PER3* VNTR with CRSWD phenotypes or diurnal preferences in our Japanese cohort. The frequency distribution of the *PER3* VNTR differs among African American, European American, Ghana Africans, Han Chinese, and Italian populations^10,14^. The data indicate that the associations between the VNTR and circadian and sleep phenotypes could differ among populations. Significant associations between the VNTR and diurnal preferences were found in the British populations^7,15^ but not in the Han Chinese populations^14^. The *PER3* VNTR may not be an appropriate genetic marker for predicting circadian and sleep phenotypes in some populations, including the Japanese population. However, a number of studies indicate close links between the *PER3* gene and circadian rhythms and sleep homeostasis^8,16,17^. Polymorphisms in the *PER3* promoter are associated with DSWPD and these polymorphisms alter *PER3* expression levels^18^. Our previous study demonstrated that a missense polymorphism in the *PER3* coding region (rs228697) is associated with N24SWD and diurnal preferences^11^. Additionally, another group has recently identified the association of rs228697 with diurnal preferences^19^. Furthermore, the PER3 protein with the rs228697 variant has been shown to be a strong repressor for CLOCK–BMAL1-induced transcription^20^. These findings suggest that the *PER3* gene could play an important role in the circadian clock system and sleep regulation.

In conclusion, our data show no significant association between the *PER3* VNTR and CRSWD phenotypes or diurnal preferences in our Japanese cohort. The results of our study imply that the *PER3* VNTR may not be a universal genetic marker for an individual’s circadian and sleep phenotypes.

## Data Availability

The relevant data from this Data Report are hosted at the Human Genome Variation Database at 10.6084/m9.figshare.hgv.2336 and 10.6084/m9.figshare.hgv.2339.
